# The Proliferation and Stemness of Peripheral Blood-Derived Mesenchymal Stromal Cells Were Enhanced by Hypoxia

**DOI:** 10.3389/fendo.2022.873662

**Published:** 2022-05-12

**Authors:** Pengzhen Wang, Pingping Zhu, Chaosheng Yu, Jian Wu

**Affiliations:** ^1^ Guangzhou Institute of Traumatic Surgery, Guangzhou Red Cross Hospital, Jinan University, Guangzhou, China; ^2^ Department of Neurology, Guangzhou Red Cross Hospital, Jinan University, Guangzhou, China; ^3^ Department of Otorhinolaryngology, Guangzhou Red Cross Medicine, Jinan University, Guangzhou, China

**Keywords:** peripheral blood-derived mesenchymal stromal cells (PBMSCs), hypoxia, HIF-1α, proliferation, stemness

## Abstract

This study aimed to address the dilemma of low peripheral blood-derived mesenchymal stromal cell (PBMSC) activity and reduced phenotype in bone or cartilage tissue engineering. Rat PBMSCs (rPBMSCs) were obtained by density gradient centrifugation, and stromal cell characteristics were confirmed by flow cytometry (FCM) and multi-differentiation potential induction experiments. Cell growth curve, viability experiments, and clone formation experiments were performed by [3-(4,5-dimethylthiazol-2-yl)-5-(3-carboxymethoxyphenyl)-2-(4-sulfophenyl)-2H-tetrazolium] (MTS) and cell counting, and the cell cycle was confirmed by cell FCM. The proliferation signal pathway and stemness-related proteins were detected by molecular methods including Western blot and real-time polymerase chain reaction. *CD73, CD90*, and *CD105* were highly expressed, and *CD14, CD19, CD34, CD45*, and *HLA-DR* were barely expressed in rPBMSCs. rPBMSCs possessed the potential to differentiate into chondrocytes, adipocytes, and osteoblasts under their respective induction conditions. Cell growth curve and viability experiments were performed under hypoxic conditions: 19% O_2_, 5% O_2_, and 1% O_2_. Specifically, 5% O_2_ accelerated the proliferation and expression of the stemness of PBMSCs. Cycle experiments proved that hypoxia promoted the cell transition from the G1 phase to the S phase. Molecular experiments confirmed that 5% O_2_ hypoxia significantly elevated the expressions of hypoxia-inducible factor 1α and β-catenin and simultaneously the expressions of cycle-related genes including *CyclinE/CDK2* and stemness-related genes including *Nanog* and *SOX2*. The appropriate concentration of hypoxia (i.e., 5% O_2_) enhanced the proliferation and stemness of rPBMSCs and increased the multidirectional differentiation potential of stromal cells. The proposed culture method could improve the viability and maintain the phenotype of rPBMSCs in cartilage or bone tissue engineering.

## Introduction

In recent years, mesenchymal stromal cells (MSCs) derived from adults have been widely used not only for bioregenerative tissue engineering but also for pathophysiological research and cell and gene therapy of bone diseases (1–4). Many studies have confirmed that bone marrow-derived MSCs are a relatively stable source, but the low yield and traumatic source of stromal cells had limited preclinical and clinical applications (5, 6). In recent literature, the applications of peripheral blood-derived MSCs (PBMSCs) in tissue engineering have attracted increasing attention because of their relatively easy collection, abundant sources, and multilineage differentiation potential (7, 8).

The committee of the International Society for Cellular Therapy standardized the criteria for defining human MSCs for basic and preclinical research. That is, cells can adhere and the MSC population must positively express *CD105*, *CD73*, and *CD90* and negatively express *CD45*, *CD34*, *CD14* or *CD11b*, *CD79a* or *CD19*, and *HLA-DR*. MSCs also have the potential to differentiate into chondrocytes, osteoblasts, and adipocytes (9). When many cells are needed for research or clinical applications, PBMSCs can take on this important task (10, 11). However, the reduced activity of PBMSCs cultured *in vitro* and phenotype loss easily limit this demand (11). Therefore, the key to cartilage tissue engineering is to provide phenotype-maintaining MSCs expanded *in vitro*. Some growth factors or physical factors, such as basic fibroblast growth factor (bFGF) (12), transforming growth factor-β (TGF-β) (13), and oxygen level (14), play a decisive role in stromal cell survival or proliferation. Thus, TGF-β, bFGF, and oxygen levels have affected stromal cell survival or proliferation (12–14). Hypoxia is a simple and easy-to-operate strategy with few side effects. The exposure of MSCs to a hypoxic environment for a moderate time could enhance cell survival characteristics and tissue repair capabilities, and this conclusion was confirmed by recent studies (15). To enhance the therapeutic effect, several studies have conducted hypoxic pretreatments in many disease-related organs and tissues, such as cardiomyocytes (16). In the literature, compared with normoxic conditions, a hypoxic condition significantly promotes MSCs to further express *Oct4*, *cMyc*, *Nanog*, and *SOX2*. Simultaneously, hypoxia-cultured MSCs exhibited a better growth trend and a higher proportion of S phase cells than normoxia-cultured MSCs (14–16).

Oxygen gradients derived from the bone marrow niche create hypoxic conditions for stromal and stem cells (17). Hypoxia strongly affects several aspects of cell biology, such as angiogenesis, innate immunity, cell proliferation, and stemness (18). The effects of hypoxia on stem cells are usually mediated by HIF-1α and HIF2α (19). The literature reported that incubation of umbilical cord derived mesenchymal stem cells (UC-derived MSCs) with different concentrations of oxygen resulted in increased cell proliferation under hypoxia. In this case, significant levels of HIF-1α could be observed in hypoxic MSCs cultured in 2.5% or 5% O_2_ (20). Hypoxia-inducible factor 1α (HIF-1α), as a pivotal transcription factor regulating stress and adaptive responses to oxygen concentration (21), usually interacts directly with numerous proteins to regulate its function (22–24). Most classically, differentiation, proliferation, angiogenesis, and migration are directly correlated with HIF-1α and β-catenin (25–27). However, how HIF-1α is expressed in PBMSCs and how it regulates the maintenance of stemness and cell proliferation remain unclear.

This study hypothesized that hypoxia could promote the proliferation and differentiation of rPBMSCs by activating the expressions of HIF-1α, β-catenin, proliferative-related genes, and stemness-related genes. Thus, this study examined the ability of rPBMSCs to proliferate and maintain MSC phenotypes under different concentrations of oxygen *in vitro* culture to explore the effects and mechanisms of hypoxia on the maintenance of rPBMSC proliferation and stemness.

## Materials and Methods

### Isolation and Culture of rPBMSCs

The animal ethics committee of Guangzhou Red Cross Hospital approved the research. Following previously published methods (28), a 3 cm × 3 cm wound was made on the back skin of the rats. The wound was disinfected every day, and the rats were provided with enough food and water to ensure their normal activities and survival. After 1 week, 0.8% pentobarbital was injected into the abdominal cavity of these animals for anesthesia. After anesthetization, approximately 5 mL of abdominal aortic blood was collected using a fine-needle approach, and the blood sample was diluted to 1:1 by PBS. Mononuclear cells (MNCs) were separated and collected with Ficoll separation solution (GBCBIO Technologies, Guangzhou, China) and centrifuged at 2000 rpm for 35 min. The middle layer was pipetted with a thin tube and washed twice with phosphate-buffered saline (PBS). MNCs (2×10^6^/mL) were seeded onto the T-25 flask with 10 mL of complete Dulbecco’s Modified Eagle Medium (Gibco, MA). The complete medium contained 1% penicillin/streptomycin (Gibco), 20 ng/mL bFGF (R&D Systems, MN), and 20% fetal bovine serum (Gibco). With 21 days of culture, the cell convergence was 80%, and the third-generation cells digested by 0.25% trypsin were used for subsequent experiments. Representative bright-field images were captured by an inverted phase-contrast microscope (Nikon ECLIPSE Ts2, Nikon).

### Immunophenotype Analysis of rPBMSCs

The cell immune phenotypes of third-generation PBMSCs (P3 PBMSCs) were identified by flow cytometry (FCM). *CD73*, *CD105*, and *CD90* (R&D Systems, US) were selected as positive markers of rPBMSCs, whereas *CD14*, *CD19*, *CD34*, *CD45*, and *HLA-DR* (BD biosciences, US) were chosen as negative markers of rPBMSCs. rPBMSCs (2×10^5^ cells/mL) were resuspended in PBS and mixed in *CD14*, *CD19*, *CD34*, *CD45*, *CD73*, *CD90*, *CD105*, and *HLA-DR* antibody solutions for 30 min, and the cell samples were then loaded on the machine for analysis.

### Cell Cycle Distribution Assay

rPBMSCs treated under normoxic and hypoxic (5% O_2_) conditions for 24 h were collected, and rPBMSCs were then fixed with 70% (V/V) ethanol overnight. Moreover, 50 μg/mL propidium iodide (PI) (Beyotime Biotechnology, Shanghai, China) was diluted by PBS solution containing 1% Triton X-100. Cells were fully infiltrated in the freshly prepared PI solution for 30 min and were analyzed by a BD FACScan flow cytometer (BD Company, CA).

### Multilineage Differentiation Potential Assay

P3 rPBMSCs were seeded into a 24-well plate at a density of 2 × 10^4^/well and cultured at 37°C in an incubator with 5% CO_2_. When the cells grow to 70% confluence, chondrogenesis induction, osteoinduction, and adipogenesis tests were performed. For chondrogenesis, cells were induced for 21 days in a chondrogenesis induction medium kit (RAXMX-90041, Cyagen Biosciences, US). The differentiation was evaluated by alcian blue staining. For osteogenesis, the cultures were induced with an osteogenesis induction medium kit (RAXMX-90021, Cyagen Biosciences, CA). After culture for 21 days, alizarin red staining was performed to evaluate the osteogenic products. For adipogenesis, cells were induced for 21 days in an adipogenesis induction medium kit (RAXMX-90031, Cyagen Biosciences). The formation of lipid vacuoles was assessed by Oil Red O staining. All images were captured under an inverted phase-contrast microscope (Nikon ECLIPSE Ts2, Nikon).

### Multilineage Differentiation Potential of rPBMSCs Cultured Under Normoxic (21% O_2_ and 5% CO_2_) or Hypoxic (5% O_2_ and 5% CO_2_) Conditions

P3 rPBMSCs were seeded into a 24-well plate at a density of 2 × 10^4^/well and cultured at 37°C in a 21% O_2_ and 5% CO_2_ incubator or a 5% O_2_ and 5% CO_2_ incubator. The induction medium and experimental procedures performed in the subsequent experiments were the same as the methods described in “Multilineage Differentiation Potential Assay.”

### Determination of the Growth Curve of rPBMSCs and the MTS Assay

P3, P5, and P6 rPBMSCs (2× 10^3^/well) were inoculated in microplates (24-well) in 5% CO_2_ incubators with a gradient concentration of oxygen at 37°C. The experiments were set up as the control group (21% O_2_ and 5% CO_2_), 19% O_2_ and 5% CO_2_ hypoxia group, 5% O_2_ and 5% CO_2_ hypoxia group, and 1% O_2_ and 5% CO_2_ hypoxia group, with three replicate wells in each group. Starting from the next day, each group of cells was digested and counted accurately with a cell counter at each time point (Days 1–8). The growth curves of each cell group were made according to the number of cells. For the MTS assay, the above-mentioned groups of cells were planted on the well plate after Day 8, and the absorbance was measured at 450 nm by a multifunctional microplate reader (BioTek, US).

### Assessment of Population Doubling Levels

After the cells reached 80-90% confluency, cells were passaged and counted. Calculate the cumulative population doubling (CPD) value using the following formula (29):


$$\frac{\text{log}10\left(\text{ cells harvested }\right)-\text{log}10\left(\text{ cells reseeded }\right)}{\log10\;(2)}$$

CPD was plotted against time in culture and performed in triplicate for each counting procedure.

### Assay for Colony Formation

Moreover, 500 rPBMSCs were cultured in 6-well plates in an incubator capable of adjusting oxygen concentration for 14 days. After fixation with paraformaldehyde for 15 min, 1 mL of crystal violet staining solution was added to the culture plate for staining clones for 30 min. Under an inverted phase-contrast microscope (Nikon ECLIPSE Ts2, Nikon), the number of clones containing more than 50 cells was counted.

### Western Blot

rPBMSCs were collected after normoxic and hypoxic (5% O_2_) treatments for 5 days, and whole-cell lysates were prepared for Western blotting in radioimmunoprecipitation assay buffer. Then, 30 μg of protein was loaded into the sample well, dispersed in the gel according to the molecular weight, and directly transferred to the poly(vinylidene fluoride) membrane (Bio-Rad, CA) in a band-to-band manner through the semi-dry transfer method. The membranes were immersed in a square dish filled with primary antibody diluent. These antibodies (HIF-1α, 36169; β-catenin, 8400; *SOX2*, 3579; *CyclinE*, 4132; *Nanog*, 8822; *GAPDH* [glyceraldehyde 3-phosphate dehydrogenase], 5174) were purchased from Cell Signaling Technology (MA), and when used, the dilution ratio was 1:1000. On the next day, membranes were incubated with secondary anti-rabbit/mouse IgG, HRP-linked antibody (#7074/7076, 1:3000, Cell Signaling Technology). The electrochemiluminescence detection mixture was used to detect the protein on the membranes. ChemiDoc XRS imaging system with Image Lab software (Bio-Rad) was used to analyze the graphs.

### Immunofluorescence Microscopy

Furthermore, 10^4^/well rPBMSCs were seeded in glass slides placed in plates treated under normoxia and hypoxia (5% O_2_) for 5 days. After sequential fixation, blocking, incubation of primary (β-catenin,1:200, 8242, Cell Signaling Technology; HIF-1α, 1:200, #36169, Cell Signaling Technology) and secondary (1:200, ZF0311, OriGene Technologies, MD) antibodies, a fluorescence microscope (Ti2-U, Nikon) was used to observe and capture pictures of interest.

### Real-Time Polymerase Chain Reaction (PCR)

The culture method of rPBMSCs was the same as with Western blot. Total RNA obtained by the TRIzol method was reversed into cDNA in the PrimeScript RT Master mix reaction system (Takara Bio, Japan). With reference to the instructions, SYBR-Green reagent (Takara Bio) was used to perform real-time PCR in triplicate in a fluorescence quantitative PCR instrument (Jena, Germany). GAPDH was used as a control to analyze relative gene expression in the 2^-ΔΔCt^ formula (30). Primer sequences are presented in **Table 1**.

**Table 1:** Sequences of primers used for gene amplification.

Genes	Forward	Reverse
GAPDH	5′-CCTGGAGAAACCTGCCAAGTAT-3′	5′-TAGCCCAGGATGCCCTTTAGT-3
β-catenin	5′- TCTGCGAACTTGCTCAGGAC -3′	5′- GAACTGGTCAGCTCAACCGA -3′
CyclinE	5′- TCCGCTTACTAGAAGTGTTTGT -3′	5′- TGTGGAAGGATAGCGATTGGG-3′
CDK2	5′- AGCTCTGCTTGCGTTCCAT -3′	5′- ACGTGCCCTCTCCAATCTTC -3′
Nanog	5′- TTAAGCTGTCTGGTCCGAGG -3′	5′- CTGAGAGAACACAGTCCGCA -3′
SOX2	5′- AGTGGTACGTTAGGCGCTTC-3′	5′- ATCGCCCGGAGTCTAGTTCT-3′
HIF-1α	5′- GGGTACGTGAGGCATGTTGA-3	5′- CCGTCGGTCAGACCAGAAAA -3′

### Statistical Analysis

Data in three replicates are presented as mean ± standard deviation. Student’s t-test or one-way analysis of variance was used to analyze differences between the two groups and among multiple groups; P < 0.05 was used to mark significant differences.

## Results

In this study, rPBMSCs were successfully isolated and cultured. Stromal cell characteristics were proved by FCM and multi-differentiation potential induction experiments. The cell growth curves of P3, P5, and P6 rPBMSCs cultured under different oxygen concentrations were drawn based on the number counted at each time point. Then, the 5% hypoxia condition that significantly promoted cell growth was used for subsequent experiments. Hypoxia (5%) significantly increased the number of stromal cell clones and the proportion of S phase cells. Real-time RCR and Western blot results revealed that hypoxia (5%) significantly promoted the expressions of HIF-1α, β-catenin, and proliferation-related and stemness-related genes.

### Characterization and Identification of rPBMSCs

**Figure 1:**
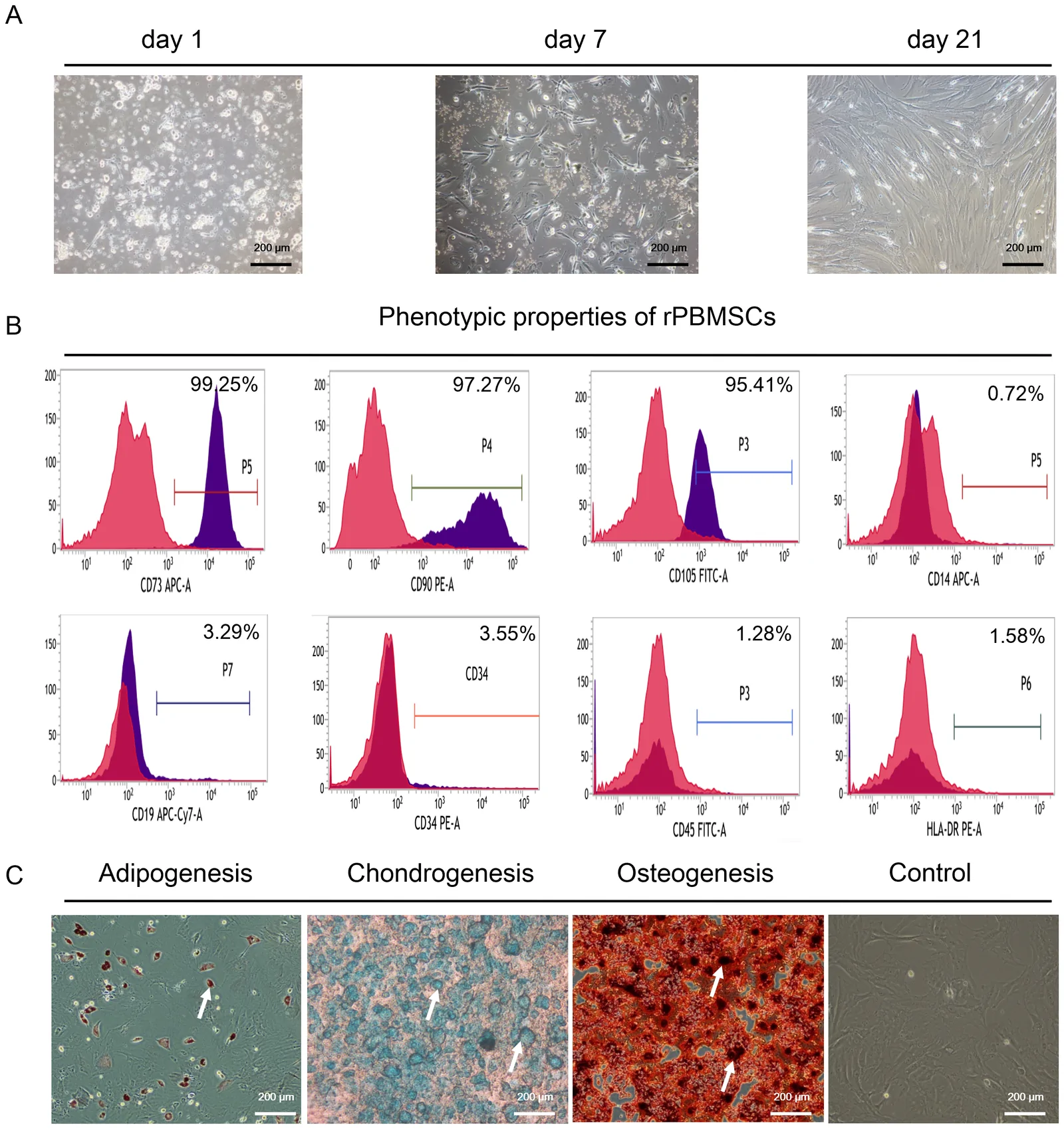
Characteristics of rPBMSCs. **(A)** Morphology of rPBMSCs cultured for 1 day, 7 days, and 21 days. **(B)** Immunocytochemical staining demonstrated a positive expression of *CD73*, *CD90*, and *CD105* and negative expressions of *CD14, CD19, CD34, CD45 CD45*, and *HLA-DR*. **(C)** Multilineage differentiation capacities of rPBMSCs. Magnification, 200×. rPBMSCs, rat peripheral blood-derived mesenchymal stromal cells.

On the day after inoculation, round or polygonal adherent cells were observed in the primary culture. After 7 days, colonies gradually formed. After approximately 16 days, the cell coverage area was 70%–80% of the bottom of the culture flask. At approximately 21 days later, the cell growth reached 100% (**Figure 1A**). Flow cytometry experiments revealed that rPBMSCs had high expression of *CD73*, *CD90*, and *CD105*, extremely low expressions of *CD14*, *CD19*, *CD34*, *CD45*, and *HLA-DR* (**Figure 1B**). Oil Red O staining indicated that rPBMSCs can differentiate into adipocytes embellished by red-stained lipid droplets. Alician blue staining demonstrated that after 21 days of induction, rPBMSCs could differentiate into chondrocytes embellished by blue-stained proteoglycans. Alizarin red staining presented that rPBMSCs could differentiate into osteoblasts embellished by red-stained bone nodules under osteogenic conditions (**Figure 1C**).

### Hypoxia Promoted rPBMSC Growth and Proliferation

**Figure 2:**
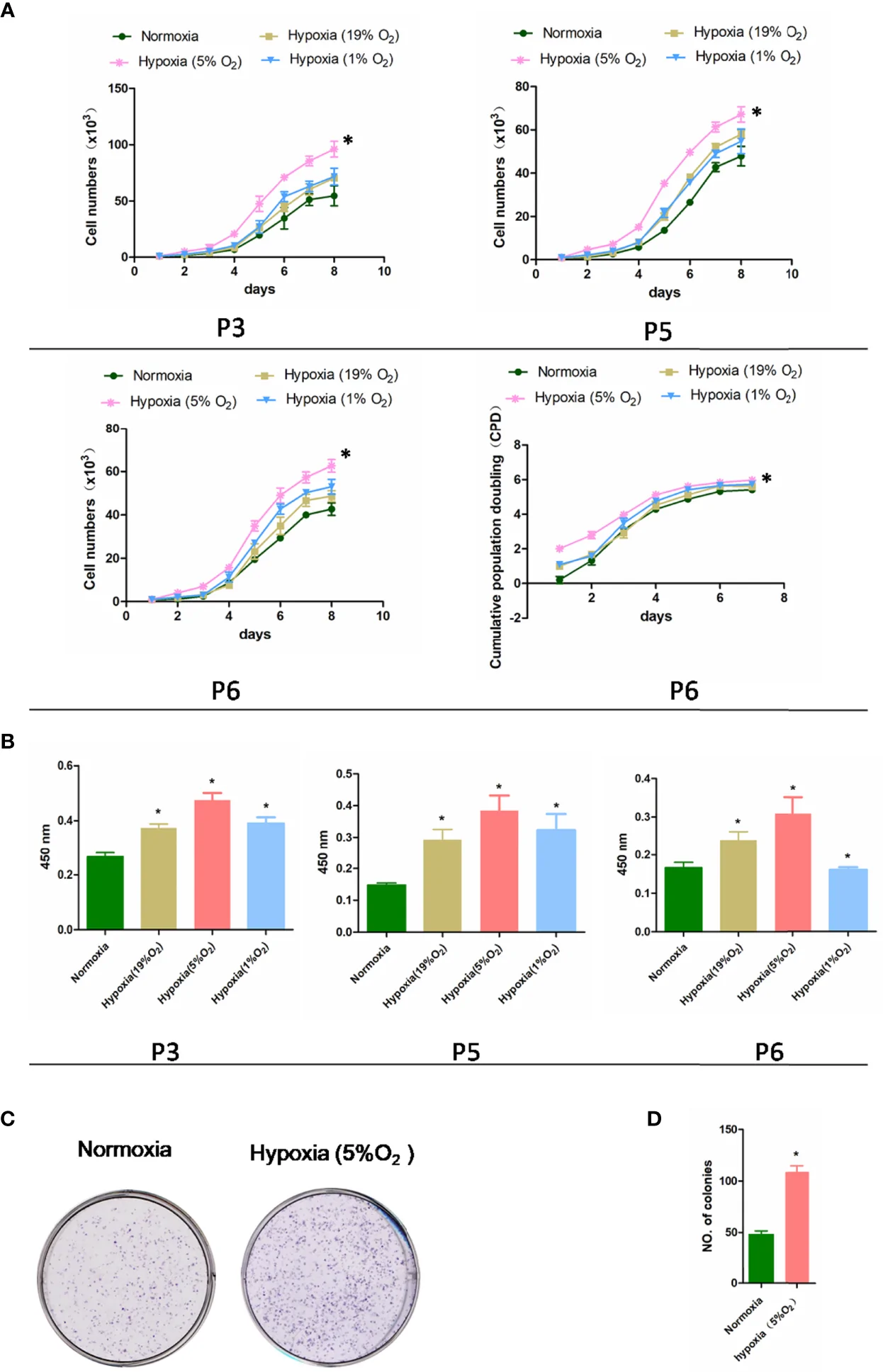
Effect of hypoxia on rPBMSC proliferation. **(A)** The cell growth curves of third-generation PBMSCs (P3 PBMSCs), fifth-generation PBMSCs (P5 PBMSCs), and sixth-generation PBMSCs (P6 PBMSCs) were drawn based on the number of cells counted at each time point, the cumulative population doubling curve of sixth-generation PBMSCs (P6 PBMSCs) was determined based on cell culture time. **(B)** Absorbance of P3, P5, and P6 rPBMSCs treated under normoxia and hypoxia (5% O_2_) at Day 8. **(C)** Hypoxia increased the number of rPBMSC colonies. **(D)** Measurement of the number of colonies in each group. All data are presented as means ± SEM. P < 0. 05; ^∗^ vs control group. rPBMSCs, rat peripheral blood-derived mesenchymal stromal cells.

The results of the experiments are displayed in **Figures 2A, B**. In the first 2 days of culture, different concentrations of O_2_ had no noticeable effects on the proliferation of third-generation PBMSCs (P3 rPBMSCs), fifth-generation PBMSCs (P5 rPBMSCs), and sixth-generation PBMSCs (P6 rPBMSCs). After 3 days, hypoxia (5% O_2_) significantly increased the number of cells and proliferation rate of P3, P5, and P6 rPBMSCs. After 8 days, the cells approached the plateau stage. At this time, the number of P3 rPBMSCs in the control group, 19% O_2_ hypoxia, 5% O_2_ hypoxia, and 1% O_2_ hypoxia groups were 55 × 10^3^, 70 × 10^3^, 96 × 10^3^, and 71 × 10^3^, respectively. Statistical analysis showed that compared with the number of P3 rPBMSCs in the control group, those in the 19% O_2_ hypoxia, 5% O_2_ hypoxia, and 1% O_2_ hypoxia groups were increased significantly (P < 0.05). Compared with 19% O_2_ hypoxia and 5% O_2_ hypoxia, 1% O_2_ hypoxia further increased the number of P3 rPBMSCs (P < 0.05). Similar to the growth curve, 5% O_2_ hypoxia significantly promoted the absorbance of P3 rPBMSCs seeded at Day 8. The CPD curve of P6 PBMSCs proved that the CPD value of PBMSCs in the 5% hypoxia group was significantly higher than that in the normoxia group. Compared with the normoxia group, the CPD values for P6 PBMSCs in the 19% O_2_ hypoxia and 1% O_2_ hypoxia groups did not change significantly on Day 7. The shape of the growth curve and viability of P5 and P6 rPBMSCs were similar with those of P3 rPBMSCs, but the amounts of rPBMSCs and optical density values of P5 and P6 on Day 8 were lower than those of P3 rPBMSCs. Based on the cell growth curve and MTT assay results, 5% O_2_ hypoxia was selected for subsequent experiments. As presented in **Figures 2C** **, D**, 5% O_2_ hypoxia significantly promoted the formation of rPBMSC colonies. The number of rPBMSC colonies in the 5% O_2_ hypoxia group was increased by 53% compared with that in the control group (P < 0.05).

### Hypoxia Promoted Cell Cycle Transition and Maintained the Trilineage Differentiation Capacity of rPBMSCs

**Figure 3:**
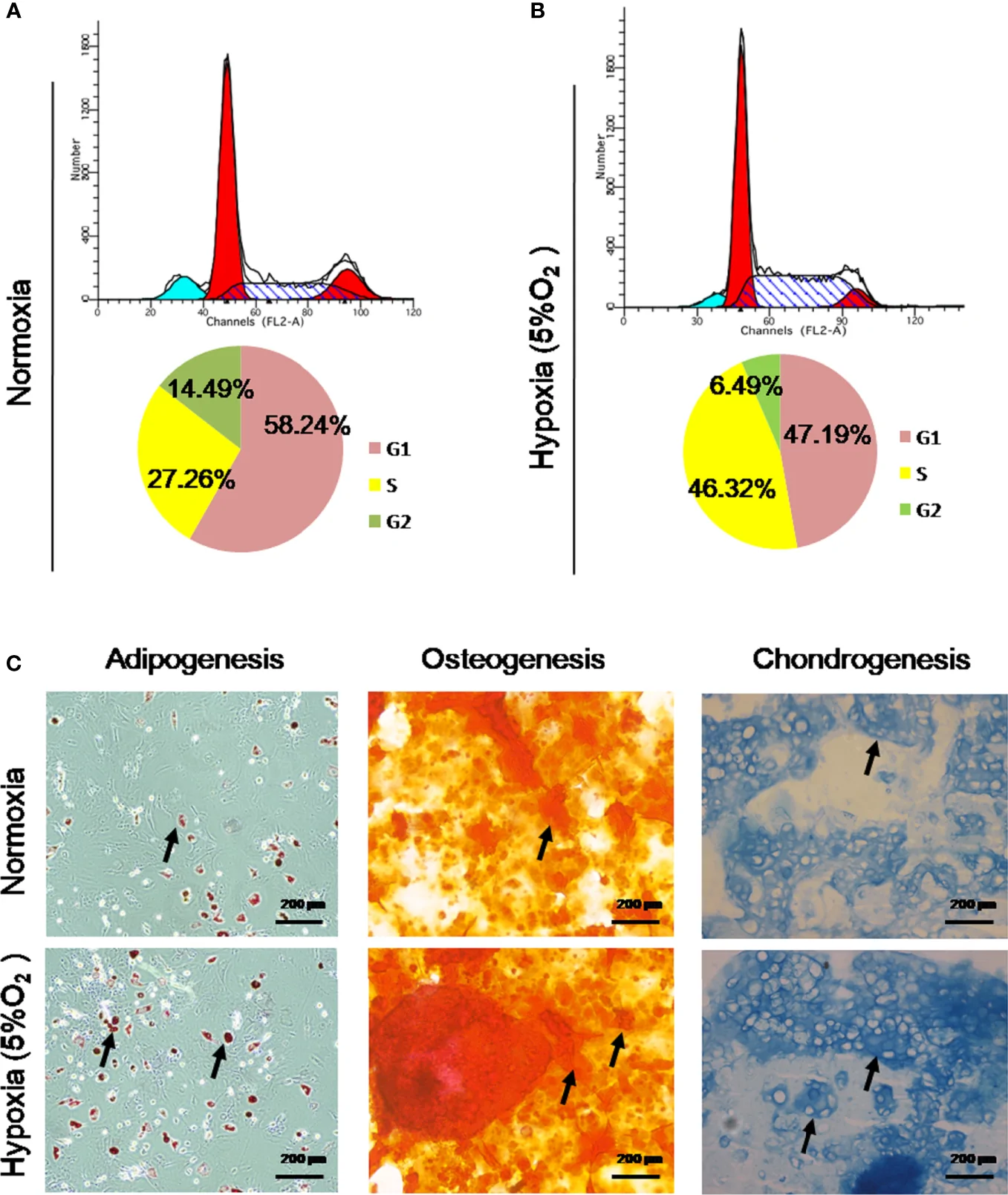
Hypoxia promoted cell cycle transition and maintained the trilineage differentiation capacity of rPBMSCs. **(A, B)** Hypoxia promoted cell cycle transition, as determined by flow cytometry. **(C)** rPBMSCs were cultured under normoxia and hypoxia (5% O_2_) for chondrogenic differentiation, osteogenic differentiation, and adipogenic differentiation for 21 days. Magnification, 200×. Arrows indicate lipid droplets, proteoglycans, and calcium nodules. rPBMSCs, rat peripheral blood-derived mesenchymal stromal cells.

FCM was used to investigate the cell cycle transition of rPBMSCs treated under normoxia and hypoxia (5% O_2_). Hypoxia exerted a significant increase and decrease in the number of S phase and G1 phase cells, respectively (**Figures 3A, B**). Moreover, 5% O_2_ hypoxia increased the percentage of rPBMSCs in the S phase from 27.26% to 46.32% (P < 0.05) and reduced the percentage of rPBMSCs in the G1 phase from 58.24% to 47.19% (P < 0.05). These data indicated that 5% O_2_ hypoxia increased the DNA synthesis and cell cycle of rPBMSC progression at the S phase. After 21 days of culture with a differentiation agent under hypoxia or normoxia, the effect of hypoxia on the pluripotency of rPBMSCs was investigated. **Figure 3C** illustrates that hypoxia increased the ability of induced cells to differentiate into three lines, including osteoblasts, chondrocytes, and adipocytes.

### Hypoxia Activated the Expression of β-Catenin and HIF-1α in rPBMSCs

**Figure 4:**
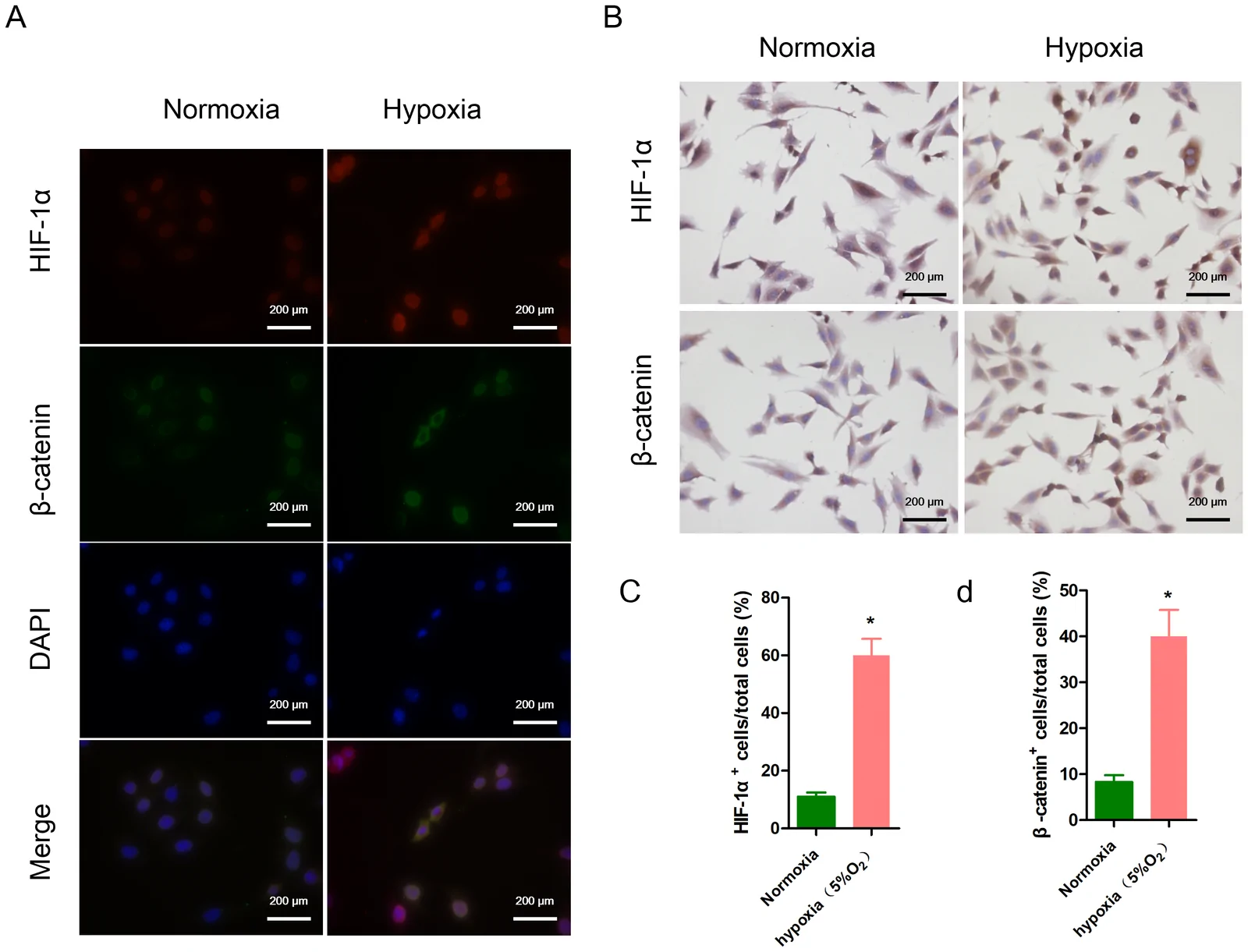
Immunoassay of HIF-1α and β-catenin in rPBMSCs under normoxia and hypoxia (5% O_2_). **(A)** Nuclear expression of HIF-1α (red) and β-catenin (green) in rPBMSCs treated under normoxia and hypoxia (5% O_2_) for 5 days. **(B)** Nuclear expression of HIF-1α (brown) and β-catenin (brown) in rPBMSCs treated under normoxia and hypoxia (5% O_2_) for 5 days. **(C, D)** Quantitative analysis of HIF-1α (brown) and β-catenin (brown) in rPBMSCs in panel **(B)**. Magnification, 200×. All data are presented as means ± SEM. P < 0. 05; ^∗^ vs control group. rPBMSCs, rat peripheral blood-derived mesenchymal stromal cells; HIF-1α, hypoxia-inducible factor 1α.

As displayed in **Figure 4A**, 5% O_2_ hypoxia significantly increased the HIF-1α (red) nuclei expression in rPBMSCs, compared with the control rPBMSCs. Simultaneously, the nuclei expression for β-catenin (green) in rPBMSCs was also upregulated significantly with 5% O_2_ hypoxia. Immunohistochemistry results (**Figure 4B**) that 5% O_2_ hypoxia stimulated the upregulation of HIF-1α and β-catenin expressions are consistent with the promotion of HIF-1α and β-catenin stabilization and nuclear translocation in immunofluorescence experiments (**Figures 4C, D**).

### Hypoxia Intensified the Expression of Cycle-Associated Genes and Stemness Genes in rPBMSCs

**Figure 5:**
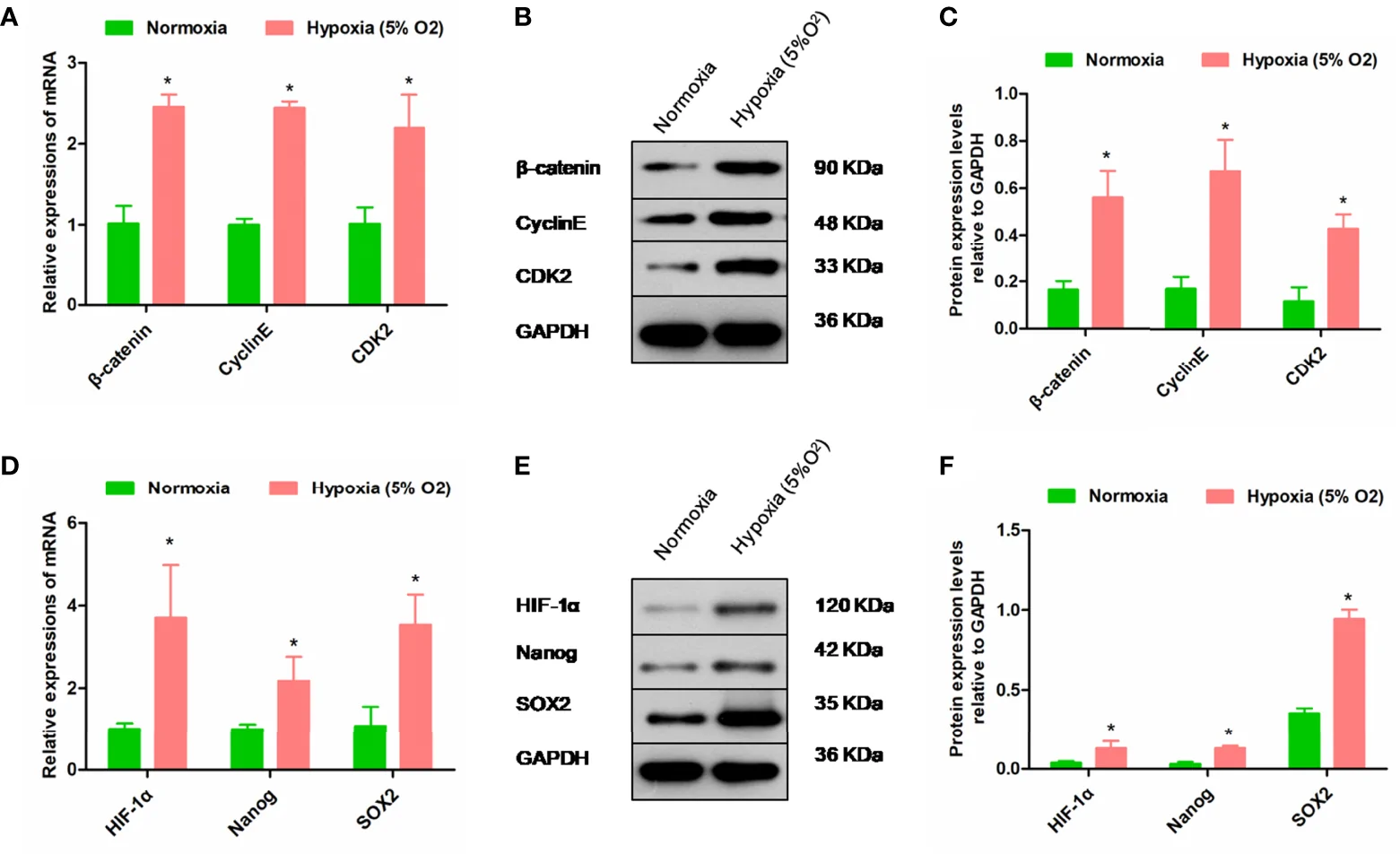
Hypoxia regulates the expressions of cycle-related and self-renewal-related molecules. **(A–C)** mRNA and protein expression levels of *β-catenin*, *Cyclin E*, and *CDK2* in rPBMSCs treated under normoxic and hypoxic (5% O_2_) conditions for 5 days. **(D–F)** mRNA and protein expression levels of *HIF-1α*, *Nanog*, and *SOX2* in rPBMSCs treated under normoxic and hypoxic (5% O_2_) conditions for 24 h. All data are presented as means ± SEM. P < 0. 05; ^∗^ vs control group. rPBMSCs, rat peripheral blood-derived mesenchymal stromal cells; HIF-1α, hypoxia-inducible factor 1α.

For the objective investigation that hypoxia regulated the self-renewal and stemness of rPBMSCs, pluripotency factors and *Cyclin E/CDK2* were primarily selected as indicators. Compared with normoxia, hypoxia significantly triggered upregulation of mRNA and protein expression for β-catenin, CDK2, and Cyclin E in rPBMSCs (**Figures 5A** **–C**). Moreover, the mRNA and protein expressions of *HIF-1α*, *Nanog*, and *SOX2* were significantly increased by hypoxia (**Figures 5D–F**).

## Discussion

As a typical representative of adult pluripotent stromal cells, PBMSCs provide various possibilities for clinical application and transformation in the field of regenerative medicine (31). PBMSCs are abundant in the body and have strong proliferation and self-renewal ability, as well as the potential to differentiate into multiple cell types (32). However, some problems and obstacles are still encountered in the application and transformation of MSCs in cartilage tissue engineering, including the reduced activity and phenotype of seed cells *in vitro (* 33). Thus, this study aimed to determine a hypoxic culture method that allows PBMSCs to maintain their ability to proliferate and self-renew during expansion *in vitro*. For this reason, this study mainly focused on three aspects, namely, (1) successful isolation, culture, and identification of rPBMSCs; (2) proliferation, phenotype maintenance, and differentiation potential of hypoxia-treated rPBMSCs; and (3) proliferation- and phenotype-related gene expressions of hypoxia-treated PBMSCs. This study proposes a culture method that is conducive to maintaining self-renewal and proliferation capabilities to ensure cell yield and long-term expansion.

In this study, rPBMSCs were successfully isolated and cultured, and third-generation rPBMSCs were selected for FCM for phenotypic identification. rPBMSCs did not express *CD45*, *CD34*, *CD14*, *CD19*, and *HLA II*, but highly expressed *CD73*, *CD90*, and *CD105*, indicating that the rPBMSCs had stromal cell performance, without other surface antigen markers (34, 35). Successfully isolated rPBMSCs positively expressed *CD90*, *CD73*, and *CD105*. Cell growth curve determination and cell cloning experiments revealed that 5% O_2_ hypoxia can significantly promote the formation of clones and the rapid proliferation of PBMSCs. Many recent studies have reported that an appropriate hypoxic condition can significantly stimulate the proliferation of MSCs and PBMSCs, which is consistent with the results of the present study. The recent studies confirmed hypoxia accelerated proliferation of PBMSCs, increased migration of PBMSCs, and reduced PBMSC differentiation into osteoblasts by increasing Notch1 expression (36). In our study, four oxygen concentration gradients were set up to more rigorously explore the effects of various oxygen concentrations on the proliferation and stemness of PBMSCs, as well as the role of HIF-1α pathway in the proliferation and stemness maintenance of PBMSCs.

Other studies have also reported that hypoxia promotes the proliferation of cord blood derived MSCs without changing the cellular immune phenotype (37). In this study, hypoxia significantly promoted the transition of PBMSCs from the G1 phase to the S phase. Since the S phase is an important stage of cell DNA synthesis (38, 39), under hypoxic conditions, PBMSCs pass the G1/S phase checkpoint and enter the DNA synthesis phase. Moreover, a study revealed that hypoxia can drive cells into the cell cycle and promote the expression of cyclins and related kinases to drive umbilical cord derived MSCs through cell cycle checkpoints, thereby promoting DNA synthesis (40).

HIF-1α is an extremely critical transcription factor, which is strongly induced during hypoxia and adapts to hypoxic tension (41). Studies have reported that HIF-1α causes cell cycle arrest in the G0/G1 phase through p27 expression (42); however, under different cell environments, HIF-1α activation can demonstrate varying results by affecting various aspects of cell biology (39). The present study presents that upregulating the expression of HIF-1α under 5% hypoxia can increase the proportion of rPBMSCs in the S phase. β-catenin is a typical cytoplasmic protein, as part of the classic Wnt signaling, which plays a role in cell adhesion (43). β-catenin coactivated LRH-1 on the cyclin E1 promoter and induced G1 cyclin-mediated cell proliferation, *Cyclin E* interacts with *CDK2* to control the G1/S phase transition (44). In this experiment, after hypoxic treatment of rPBMSCs, the transition of cells from the G1 phase to the S phase and the activation of *Cyclin E/CDK2* indicated that hypoxia may regulate the cell cycle position to control the self-renewal of rPBMSCs. Similar reports have suggested that HIF-1α promotes cell vitality and proliferation of MSCs (45, 46).

The expression of stemness markers was observed under hypoxic conditions. The significant role of low oxygen in altering the characteristics of various types of stromal cells was previously investigated (47). A study suggested the upregulation of stemness genes such as *OCT4* and *Nanog* of BMSCs cultured in 1% oxygen (48). Similar results were observed in the present study, where the expressions of *Nanog* and *SOX2* of rPBMSCs under 5% oxygen conditions were promoted, indicating that the stemness of rPBMSCs was enhanced by a hypoxic environment. The inhibition of senescence of MSCs suggests the increased expression of pluripotency markers (49). In this study, hypoxia-treated rPBMSCs demonstrated a higher differentiation potential, including cartilage, osteogenic, and adipogenic potentials compared with normoxia-treated cells. However, the control of hypoxia on stromal cells involves transcription factors such as HIF-1α and β-catenin. However, further research is needed to understand how HIF-1α and β-catenin regulate and interact with each other.

## Conclusion

**Figure 6:**
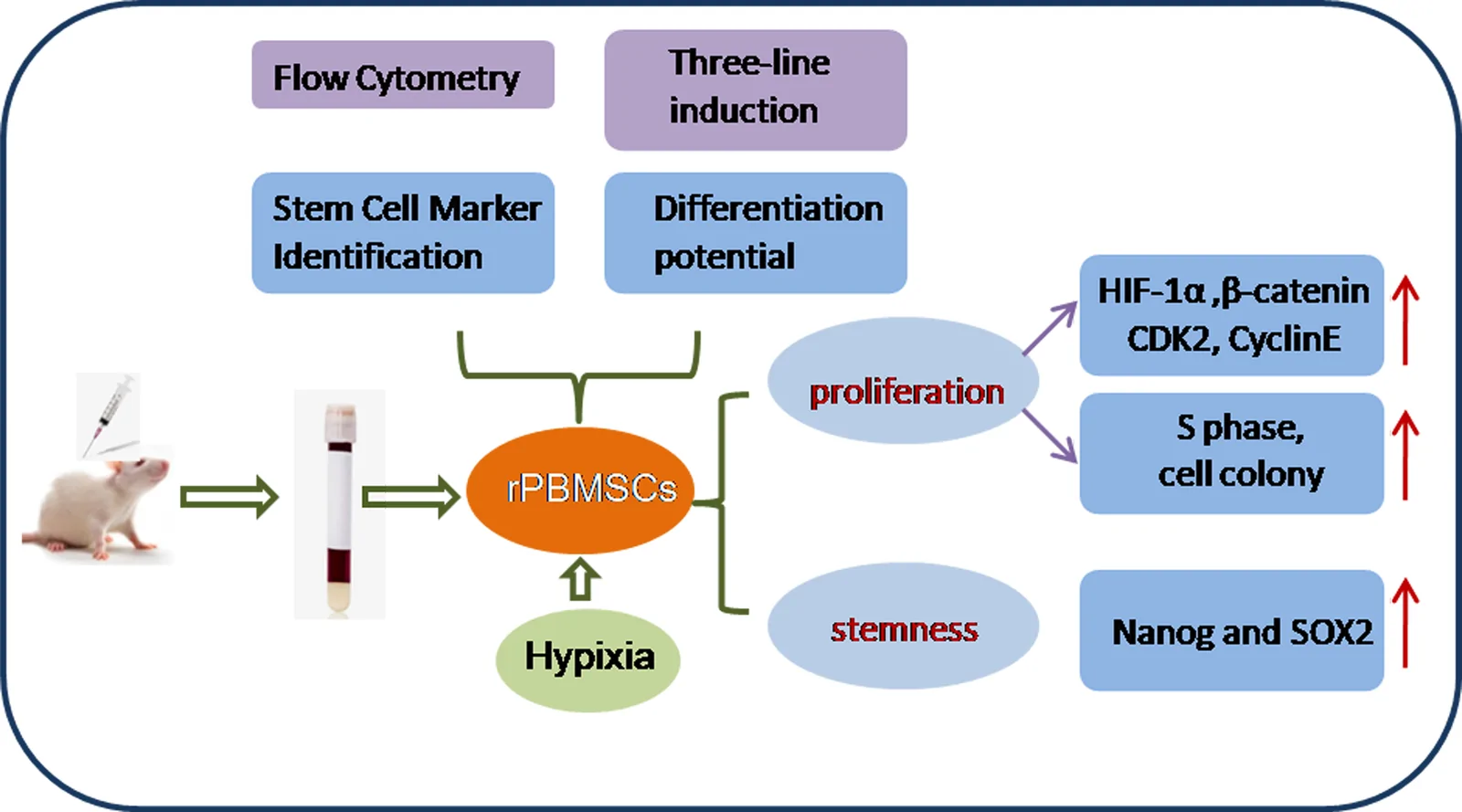
Mechanism of hypoxia-promoted proliferation and stemness of rPBMSCs.

In summary, the stemness, proliferation, and self-renewal potential of hypoxia-treated rPBMSCs were enhanced. Therefore, conditional hypoxia (5%) culture can be used as a convenient strategy to maintain the function of rPBMSCs. The general process and conclusions of this study are clearly illustrated in **Figure 6**.

## Data Availability Statement

The original contributions presented in the study are included in the article/supplementary material. Further inquiries can be directed to the corresponding author.

## Ethics Statement

This animal study was reviewed and approved by animal ethics committee of Guangzhou Red Cross Hospital.

## Author Contributions

Study design: PW; Data collection: PZ; Data analysis: CY and JW; Interpretation of data: PW; Draft manuscript: PW; Review manuscript: PW. All authors contributed to the article and approved the submitted version.

## Funding

This work was supported by the Medical Science and Technology Research Foundation of Guangdong (A2021335, PW), Traditional Chinese Medicine Bureau of Guangdong Province (20222166, PW), and Guangdong Provincial Basic and Applied Basic Regional Joint Fund (2020A1515110009, PZ).

## Correction note

A correction has been made to this article. Details can be found at: 10.3389/fendo.2026.1937220.

## Conflict of Interest

The authors declare that the research was conducted in the absence of any commercial or financial relationships that could be construed as a potential conflict of interest.

## Publisher’s Note

All claims expressed in this article are solely those of the authors and do not necessarily represent those of their affiliated organizations, or those of the publisher, the editors and the reviewers. Any product that may be evaluated in this article, or claim that may be made by its manufacturer, is not guaranteed or endorsed by the publisher.
