# Correction: The proliferation and stemness of peripheral blood-derived mesenchymal stromal cells were enhanced by hypoxia

**DOI:** 10.3389/fendo.2026.1937220

**Published:** 2026-09-30

**Authors:** Pengzhen Wang, Pingping Zhu, Chaosheng Yu, Jian Wu

**Affiliations:** 1 Guangzhou Institute of Traumatic Surgery, Guangzhou Red Cross Hospital, Jinan University, Guangzhou, China; 2 Department of Neurology, Guangzhou Red Cross Hospital, Jinan University, Guangzhou, China; 3 Department of Otorhinolaryngology, Guangzhou Red Cross Medicine, Jinan University, Guangzhou, China

**Keywords:** peripheral blood-derived mesenchymal stromal cells (PBMSCs), hypoxia, HIF-1α, proliferation, stemness

There were mistakes in **Figures 1C**, **4A** as published. In **Figure 1C**, the representative image of the Adipogenesis staining was uploaded incorrectly. In **Figure 4A**, the representative image of the Merge of HIF-1α, β-catenin and DAPI staining was uploaded incorrectly. The errors in the above Figures did not affect any of the quantitative results, statistical significance, or core conclusions in the published research. The corrected **Figures 1**, **4** appear below.

**Figure 1:**
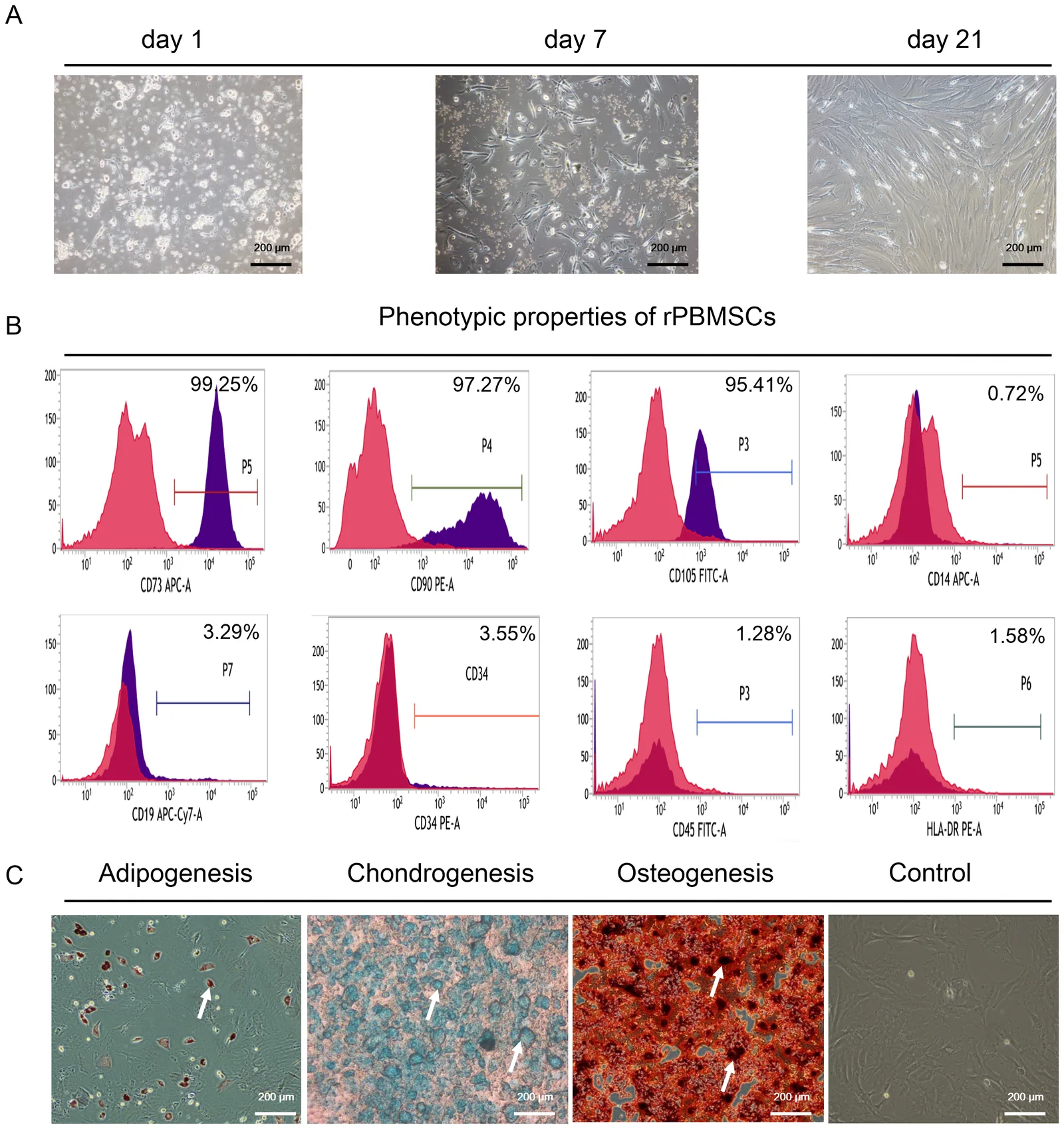
Characteristics of rPBMSCs. **(A)** Morphology of rPBMSCs cultured for 1 day, 7 days, and 21 days. **(B)** Immunocytochemical staining demonstrated a positive expression of CD73, CD90, and CD105 and negative expressions of CD14, CD19, CD34, CD45 CD45, and HLA-DR. **(C)** Multilineage differentiation capacities of rPBMSCs. Magnification, 200×. rPBMSCs, rat peripheral blood-derived mesenchymal stromal cells.

**Figure 4:**
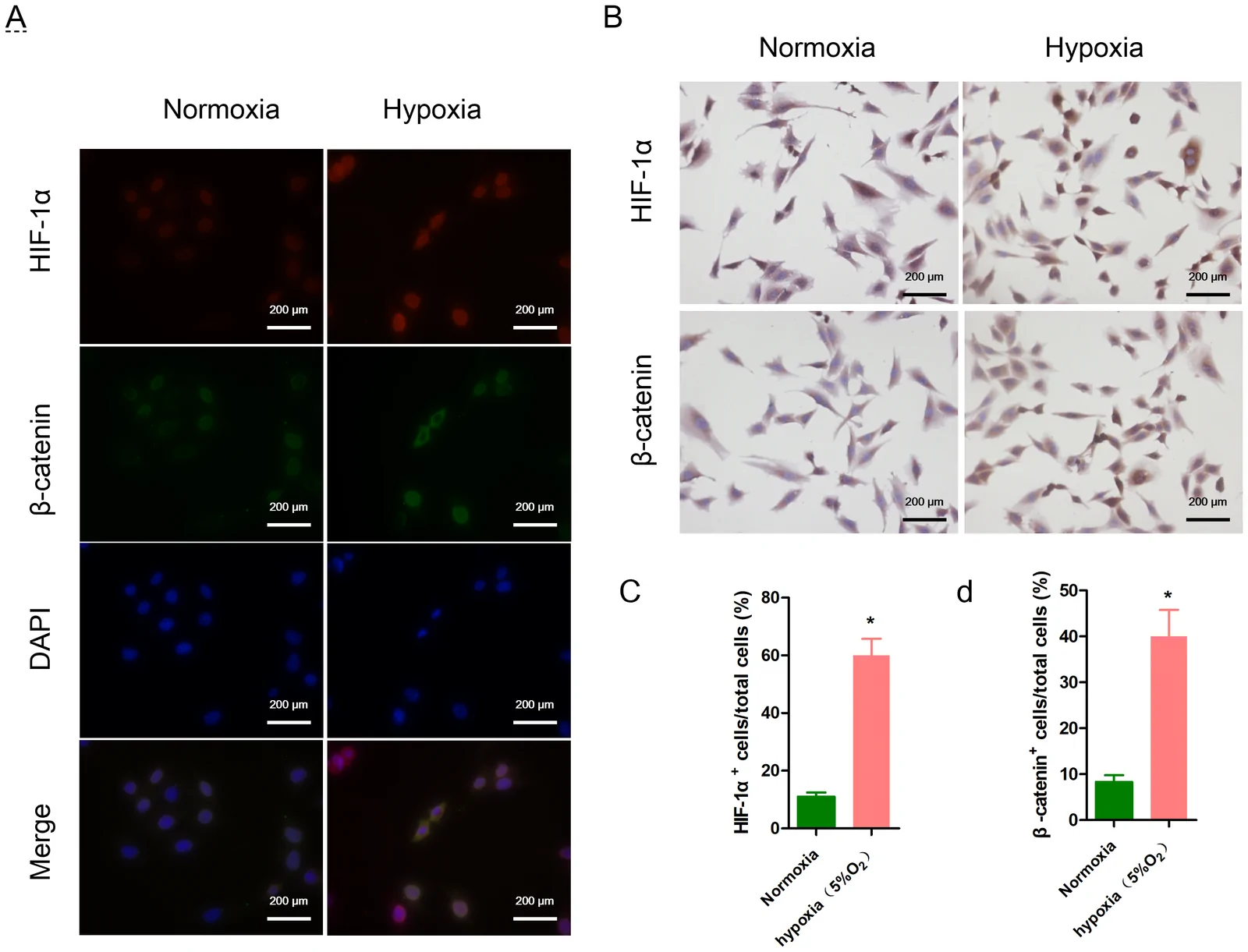
Immunoassay of HIF-1a and b-catenin in rPBMSCs under normoxia and hypoxia (5% O_2_). **(A)** Nuclear expression of HIF-1a (red) and b-catenin (green) in rPBMSCs treated under normoxia and hypoxia (5% O_2_) for 5 days. **(B)** Nuclear expression of HIF-1a (brown) and b-catenin (brown) in rPBMSCs treated under normoxia and hypoxia (5% O2) for 5 days. **(C, D)** Quantitative analysis of HIF-1a (brown) and b-catenin (brown) in rPBMSCs in panel **(B)**. Magnification, 200×. All data are presented as means ± SEM. P < 0. 05; ∗ vs control group. rPBMSCs, rat peripheral blood-derived mesenchymal stromal cells; HIF-1a, hypoxia-inducible factor 1a.

The original version of this article has been updated.

